# Retraction: The novel tumor suppressor gene ZNF24 induces THCA cells senescence by regulating Wnt signaling pathway resulting in inhibition of THCA tumorigenesis and invasion

**DOI:** 10.3389/fonc.2024.1443826

**Published:** 2024-06-18

**Authors:** Frontiers Editorial Office

Please find the retraction statement below:

Following publication, the authors contacted the Editorial Office to request the retraction of the cited article, stating that they discovered a fault in their experimental design that rendered the conclusion unreliable. An investigation was conducted in accordance with Frontiers’ policies that confirmed this; therefore, the article has been retracted.

This retraction was approved by the Chief Editors of Oncology and the Chief Executive Editor of Frontiers. The authors agree to proceed with the retraction and sincerely regret any inconvenience this may have caused to the reviewers, editors and readers of Frontiers in Oncology.

